# Effects of continuous infusion of phenylephrine vs. norepinephrine on parturients and fetuses under LiDCOrapid monitoring: a randomized, double-blind, placebo-controlled study

**DOI:** 10.1186/s12871-020-01145-0

**Published:** 2020-09-07

**Authors:** Kunpeng Feng, Xiaohua Wang, Xuexin Feng, Jinfeng Zhang, Wei Xiao, Fengying Wang, Qi Zhou, Tianlong Wang

**Affiliations:** 1 Department of Anesthesiology, Xuanwu Hospital, Capital Medical University, Beijing, 100053 China; 2 National Clinical Research Center for Geriatric Disorders, Beijing, 100053 China; 3 Department of Anesthesiology, Shanxi provincial People’s Hospital, Taiyuan, 030000 Shanxi China; 4 Department of Obstetrics, Xuanwu Hospital, Capital Medical University, Beijing, 100053 China

**Keywords:** Cesarean delivery, In-term parturient, LiDCOrapid system, Norepinephrine, Phenylephrine, Spinal anesthesia

## Abstract

**Background:**

Hypotension following spinal anesthesia (SA) during cesarean delivery (CD) occurs commonly and is related with maternal and fetal complications. Norepinephrine infusion is increasingly used for prevention of post-SA hypotension; however, its effects as compared to the traditional phenylephrine infusion remain unclear. This study aimed to compare the effects of phenylephrine and norepinephrine administered as continuous infusion during elective CD on maternal hemodynamic parameters and maternal and fetal outcomes.

**Methods:**

This prospective, single-center, randomized, controlled study included 238 consecutive term parturients who underwent CD from February 2019 to October 2019. They were randomized to receive continuous infusion of 0.25 μg/kg/min phenylephrine, 0.05 μg/kg/min norepinephrine, or placebo. Hemodynamic monitoring was performed at 10 time points using LiDCOrapid. We analyzed umbilical vein (UV), umbilical artery (UA), and peripheral vein (PV) blood gas indexes and recorded intraoperative complications.

**Results:**

In phenylephrine group, the systolic blood pressure (SBP) remain during the whole operation. Compared to the control group, phenylephrine, but not norepinephrine, significantly increased the systemic vascular resistance (SVR) to counteract the SA-induced vasodilatation, 3 min following norepinephrine/phenylephrine/LR administration (T4): 957.4 ± 590.3 vs 590.1 ± 273.7 (*P* < 0.000001); 5 min following norepinephrine/phenylephrine/LR administration (T5): 1104 ± 468.0 vs 789.4 ± 376.2 (*P* = 0.000002). at the time of incision (T6): 1084 ± 524.8 vs 825.2 ± 428.6 (*P* = 0.000188). Parturients in the phenylephrine group had significantly lower UV (1.91 ± 0.43) (*P* = 0.0003) and UA (2.05 ± 0.61) (*P* = 0.0038) lactate level compared to controls. Moreover, the UV pH value was higher in the phenylephrine than in the control group7.37 ± 0.03(*P* = 0.0013). Parturients had lower incidence of nausea, tachycardia, hypotension in phenylephrine group.

**Conclusions:**

In this dataset, continuous phenylephrine infusion reduced the incidence of SA-induced hypotension, ameliorated SVR, while decreasing overall maternal complications. Phenylephrine infusions are considered the better choice during CD because of the significant benefit to the fetus.

**Trial registration:**

Clinicaltrial.gov Registry, NCT03833895, Registered on 1 February 2019.

## Background

Spinal anesthesia (SA) is the standard and preferred mode of care for elective cesarean delivery (CD) [1], but SA can negatively affect the parturient or the fetus by reducing the placental perfusion [2]. SA-induced maternal hemodynamic fluctuations during CD can invoke nausea and vomiting, cardiovascular collapse, massive hemorrhage, unconsciousness with resulting pulmonary aspiration, or, in extreme cases, cardiorespiratory arrest [3, 4]. According to recent studies, SA-induced hypotension occurs in 80% of all parturients and nearly 60% of in-term parturients during CD without prophylactic use of vasopressors due to sympathetic blockade by the anesthesia [5, 6]. Thus, obstetric anesthetists increasingly opt for prophylactic vasopressor use for routine prevention of post-SA hypotension during CD [7]. In recent years, the optimization of hemodynamics, particularly post-SA hypotension during CD, remains the critical management challenge for anesthesiologists.

Phenylephrine, an α-adrenergic agonist and a vasopressor of choice in obstetric anesthesia, is sometimes associated with maternal cardiac depression or reflex bradycardia. This cardiac depressant effect limits its use in parturients with cardiac comorbidities. Norepinephrine, a potent α-adrenergic agonist with weak β-adrenergic agonistic activity, is associated with a lower incidence of maternal bradycardia. Thus, recently, norepinephrine is considered a potential vasopressor of choice during CD at a maintenance dose of 0.05 μg/kg/min [8]. These pharmacologic properties make norepinephrine and phenylephrine attractive choices as vasopressors in CD. However, new evidence points to post-SA hypotension reversal by phenylephrine without significant maternal bradycardia [9]. In addition, prophylactic use of phenylephrine at 0.25 μg/kg/min results in better neonatal outcomes and reduced maternal mortality [10]. The choice for phenylephrine has been reported to be more beneficial for parturients [11, 12] Nonetheless, comparative studies of these two drugs for continuous infusion are limited, and evidence on the optimum vasopressor choice is lacking.

Therefore, this study aimed to compare the effects of phenylephrine and norepinephrine administered as continuous infusion during elective CD on 1) maternal hemodynamic parameters using noninvasive LiDCOrapid™; and 2) maternal and fetal outcomes based on umbilical vein (UV), umbilical artery (UA), and maternal peripheral vein (PV) blood gas indexes.

## Methods

### Ethical considerations

This study was approved by the Capital Medical University Institutional Review Board on January 23, 2019 (IRB # 2019–058). Written informed consent was obtained from all participants. The study was registered at ClinicalTrials.gov (http://clinicaltrials.gov; NCT-03833895) on February 1, 2019. Participant recruitment was performed from February 2019 to October 2019. Our methodology followed the international guidelines for randomized clinical studies according CONSORT Guidelines.

### Study design and participants

This was a prospective, single- center, randomized, controlled clinical study conducted from February 2019 to October 2019 in the Xuanwu Hospital, Beijing, China. Parturient meeting the following inclusion criteria were recruited: 1) healthy singleton pregnancy; 2) scheduled elective CD under combined spinal-epidural anesthesia (CSEA); 3) American Society of Anesthesiologists physical status I/II; and 4) age between 20 and 45 years. The exclusion criteria were as follows: 1) history of mental disorder, epilepsy, or other central nervous system disease; 2) tricyclic or imipramine antidepressant use; 3) preexisting or pregnancy-induced hypertension; 4) lumbar injury; 5) severe hypovolemia; 6) allergy or history of hypersensitivity to vasopressors; 7) body mass index > 40 kg/m^2^; and 8) infection at the puncture site.

### Randomization and blinding

Randomization was performed using computer-generated randomized numbers and allocation concealment was ensured using sequentially numbered opaque sealed envelopes. An anesthesiologist not involved in parturient care was responsible for opening the envelopes and preparing the study medicine.

The study medicine and sealed wrapping instructions were delivered to the operating room before the time of CD. The study medicine was prepared in 50 mL syringes containing phenylephrine, norepinephrine, or placebo, marked with a randomization number. The dose of each medicine was calculated according to the participant’s standard weight, defined as the actual height minus 110 cm [13], and then the medicine was diluted to 50 mL at different concentrations. The three groups were infused in the same speed at 20 ml/h. Anesthesiologists involved in infusion of the medicine or parturient care were blinded to the group allocation. Randomization codes were not revealed to the blinded anesthesiologists until all measurements and calculations had been entered into the database and statistical methods had been specified.

### Anesthesia protocol

On arrival in the operating room, standard monitoring was initiated, including noninvasive blood pressure (BP) measurement, heart rate (HR) measurement, pulse oximetry, and electrocardiography. Patients were asked to rest still for 5 min. Subsequently, hemodynamic parameters were measured thrice at 2-min intervals, and the mean value was considered the baseline. Next, venous access was established using a 16-gauge intravenous (IV) cannula and 10 mL/kg lactated Ringer’s solution (LR) was infused in all groups before CSEA.

CSEA was performed with the patient in the right lateral position using 0.5% bupivacaine (7.5 mg, 1.5 mL, isobaric, 1.0 mL/10 s) injected into the subarachnoid space at the L2–L3 interspace. An epidural catheter was inserted cephalad for a rescue SA. Immediately after anesthesia induction, patients were placed in the supine position with 15° left lateral tilt. The sensory block level before surgical incision was T4.

Intraoperatively, maintenance LR (3 ml/kg/h) was provided for all groups according to the parturients’ standard weight. Additionally, parturients received a continuous infusion of the study drug according to the group allocation. After delivery of the fetus, a bolus of 5 IU oxytocin was administered IV followed by a slow infusion of another 5 IU over the remainder of the operation in all three groups.

### Interventions

In the phenylephrine group, parturients received a continuous infusion of phenylephrine at the rate of 0.25 μg/kg/min according to their standard weight [14]. In the norepinephrine group, parturients received a continuous infusion of norepinephrine at the rate of 0.05 μg/kg/min according to their standard weight [8]. In the control group, parturients received a continuous infusion of LR as the same speed.

Hypotension was defined if the systolic BP (SBP) reduced by 30% relative to the baseline value or an absolute SB*P* value of < 100 mmHg. The time interval for BP measurement was set at 3 min. The shortest interval for vasopressor administration was every 1 min. In case of severe hypotension (SBP reduced by more than 30% relative to the baseline value), additional bolus of vasopressor was given; 25 μg of phenylephrine in the phenylephrine group or 4 μg of norepinephrine in the norepinephrine group. In the control group, additional bolus of 4 μg norepinephrine was administered in case of hypotension combined with a HR > 60 bpm and additional bolus of 25 μg phenylephrine was administered in case of hypotension combined with a HR < 60 bpm. In this study, 0.5 mg atropine was administered continually for 3 min only in case of simple bradycardia (HR < 50 bpm). The vasopressor infusion was stopped if the SBP increased to > 150 mmHg for over 3 min.

### Outcome measurement

LiDCOrapid Pulse Contour Analysis System (LiDCO Ltd., London, UK) was used in all three groups to measure the hemodynamic parameters at each time point. The hemodynamic parameters included stroke volume (SV), cardiac output (CO), systemic vascular resistance (SVR), SBP, diastolic blood pressure (DBP), mean arterial pressure (MAP), and HR. All parameters were measured at baseline (T1), at the time of spinal injection (T2), at placement in supine position (T3), 3 min following norepinephrine/phenylephrine/LR administration (T4), 5 min following norepinephrine/phenylephrine/LR administration (T5), at the time of incision (T6), immediately after fetus delivery (T7), at the time of placental expulsion (T8), 5 min after placental expulsion (T9), and at discharge to the postoperative unit (T10).

Blood samples were taken from the UA, UV, and PV for analysis by the blood gas analyzer (Radiometer ABL800 FLEX analyzer, Radiometer A/S, Copenhagen, Denmark) immediately after delivery. The measured parameters included oxygen partial pressure (PO_2_), oxygen saturation (SO_2_), carbon dioxide partial pressure (PCO_2_), glucose and lactate levels, base excess (BE), pH, and anion gap (AG). Intraoperative fluid input and output were recorded. The postoperative incidence of maternal complications, such as hypotension, tachycardia, bradycardia, nausea and vomiting, breathing difficulty, and dizziness was also recorded.

The primary outcome of the study was the SBP as important one of hemodynamic parameters in each group at different time point. The secondary outcomes included hemodynamic parameters (DBP, MAP, HR, SV, CO, SVR), the blood gas indices (PO_2,_ SO_2,_ PCO_2,_ BE, pH, AG) in UV, UA, and PV blood samples, and the incidence of complications.

### Sample size calculation

In our pilot study (*n* = 20), the increase of systolic blood pressure (SBP) in the norepinephrine and phenylephrine compare with control groups were ∆31 mmHg and ∆20 mmHg respectively. Using PASS 15.0, a sample size of 71 in the phenylephrine group and 70 in the norepinephrine group was required for α (Type I error) of 0.05 and β (Type II error) of 0.2. Considering a 10% withdrawal rate, the sample size was calculated at 79 per group.

### Statistical analysis

Statistical analyses were performed using SPSS (version 22.0, SPSS Inc., Chicago, IL, USA). Categorical data were expressed as number of episodes/participants counts and compared among the three groups by the Chi-squared test. Intergroup comparisons of the mean values of parameters and the mean variations using the Tukey Kramer multiple comparison test. In the time-series data in each group was determined using one-way repeated measures ANOVA during the whole operation. All data were analyzed by the Shapiro-Wilk test for normality of distribution. Normally distributed quantitative variables were presented as means ± standard deviation. A *P* value of < 0.05 was considered statistically significant.

## Results

### Demographic data

**Fig. 1:**
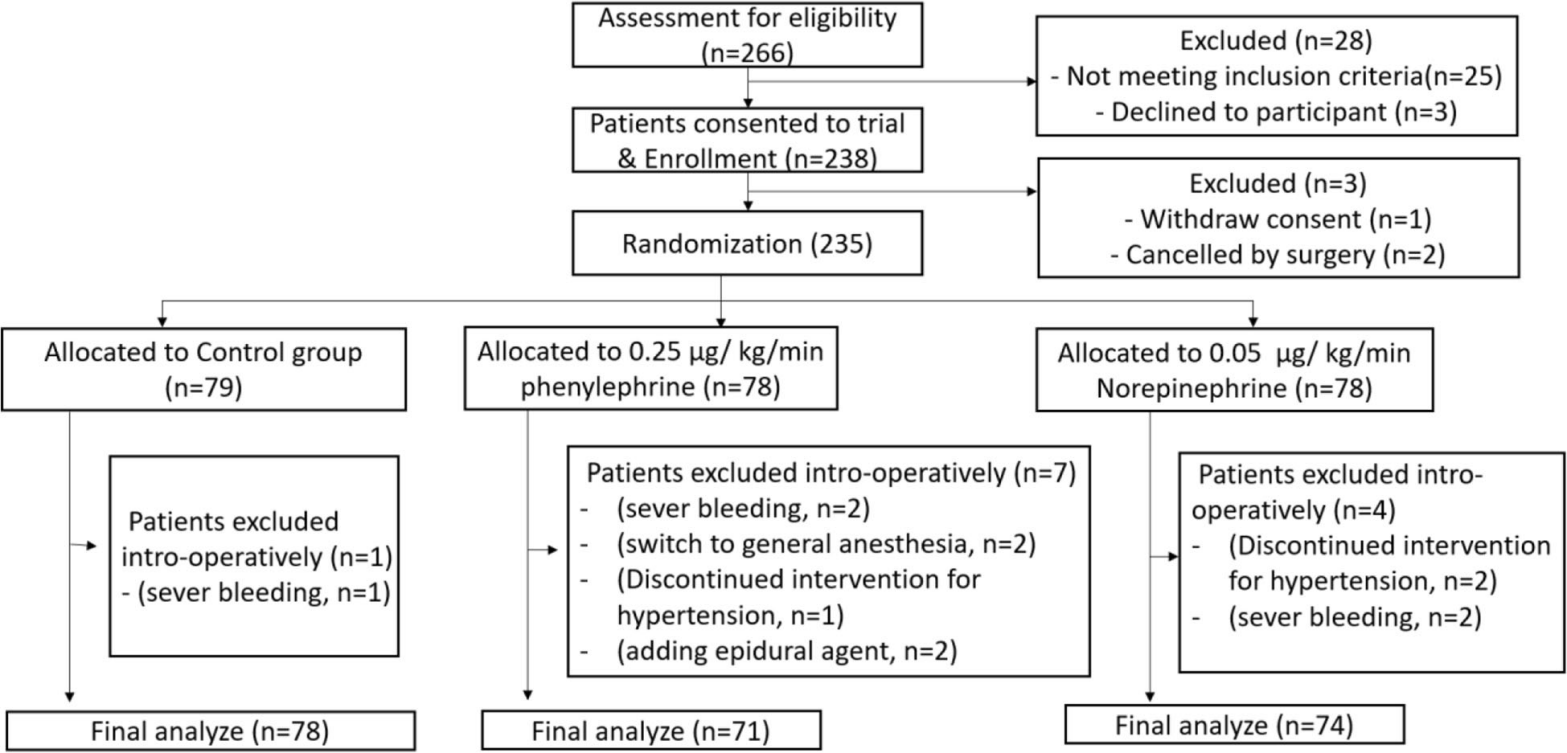
Participant flowchart

Of the 266 recruited parturients, 28 were excluded, 238 were included in the study, and 235 successfully completed the study (Fig. 1). The parturient’ s demographics (age, weight, height, BMI) and baseline of the parameter (SBP, DBP, MAP, HR, CO, SV, SVR) were similar in all three groups (Table 1). The median sensory block height at skin incision reached T4 in all three groups. The urine output, amount of blood loss, and the total volume of infusion were also similar in all groups. There was no significant difference in the duration of delivery, anesthesia, and operation, and the APGAR score among the three groups (Table 2).

**Table 1:** Parturient demographics and baseline characteristics of the phenylephrine, norepinephrine and control group

Variables	Control group (*n* = 78)	Phenylephrine group (*n* = 71)	Norepinephrine group (*n* = 74)	F value	*P* value
Age, years	34.04 (4.80)	33.34 (3.77)	33.70 (4.26)	0.4904	0.6130
Weight, kg	77.77 (12.28)	75.36 (8.43)	76.09 (11.41)	0.9642	0.3829
Height, cm	162.41 (5.72)	161.70 4.27 (4.27)	161.77 (5.46)	0.4253	0.6541
BMI, kg/m^2^	29.49 (4.52)	28.79 (2.59)	29.06 (3.92)	0.6635	0.5161
Baseline CO, L/min	8.42 (2.46)	7.87 (2.58)	8.43 (2.53)	1.182	0.3087
Baseline SVR, dyn s m^2^/ cm^5^	968.37 (344.23)	968.37 (269.26)	971.17 (340.18)	0.045	0.9561
Baseline SV, ml	95.83 (25.14)	91.26 (26.87)	93.85 (24.46)	0.6079	0.5454
Baseline SBP, mmHg	120.19 (11.72)	117.14 (9.66)	120.64 (12.38)	2.054	0.1306
Baseline DBP, mmHg	68.44 (13.04)	66.49 (9.79)	69.54 (11.98)	1.245	0.2901
Baseline MAP, mmHg	81.28 (15.81)	78.68 (11.31)	81.59 (18.59)	0.7657	0.4662
Baseline Heart rate, beats/min	87.38 (12.81)	87.41 (11.57)	91.16 (10.54)	2.797	0.0631

*BMI* Body Mass Index, Data are expressed as mean (SD); *SD* Standard deviation. *0.25 μg/kg/min phenylephrine vs. control group, *P* < 0.05; #0.05 μg/kg/min norepinephrine vs. control group, *P* < 0.05; †0.25 μg/kg/min phenylephrine vs. 0.05 μg/kg/min norepinephrine group, *P* < 0.05 based on ANOVA

**Table 2:** Intraoperative characteristics of the phenylephrine, norepinephrine and control group

Variables	Control group (*n* = 78)	Phenylephrine group (*n* = 71)	Norepinephrine group (*n* = 74)	F value	*P* value
Bleeding, ml	249.36 (64.71)	236.62 (68.12)	248.65 (78.06)	0.9075	0.405
Urine output, ml	214.23 (76.73)	211.97 (68.38)	214.19 (84.20)	0.0206	0.980
Total volume of infusion, ml	847.09 (230.77)	786.22 (135.33)	791.89 (161.97)	2.587	0.078
Operation duration, min	44.76 (12.43)	42.87 (11.19)	42.08 (8.11)	1.244	0.290
Anesthesia duration, min	71.60 (16.53)	70.21 (15.85)	72.14 (11.51)	0.3249	0.723
Delivery duration, min	5.04 (2.18)	5.21 (2.03)	5.12 (1.97)	0.1301	0.878
APGAR score	9.97 (0.16)	10 (0)	9.99 (0.12)	0.9158	0.402

Data are expressed as mean (SD); *0.25 μg/kg/min phenylephrine vs. control group, *P* < 0.05; #0.05 μg/kg/min norepinephrine vs. control group, *P* < 0.05; †0.25 μg/kg/min phenylephrine vs. 0.05 μg/kg/min norepinephrine group, *P* < 0.05 based on ANOVA

### Hemodynamic parameters

**Fig. 2:**
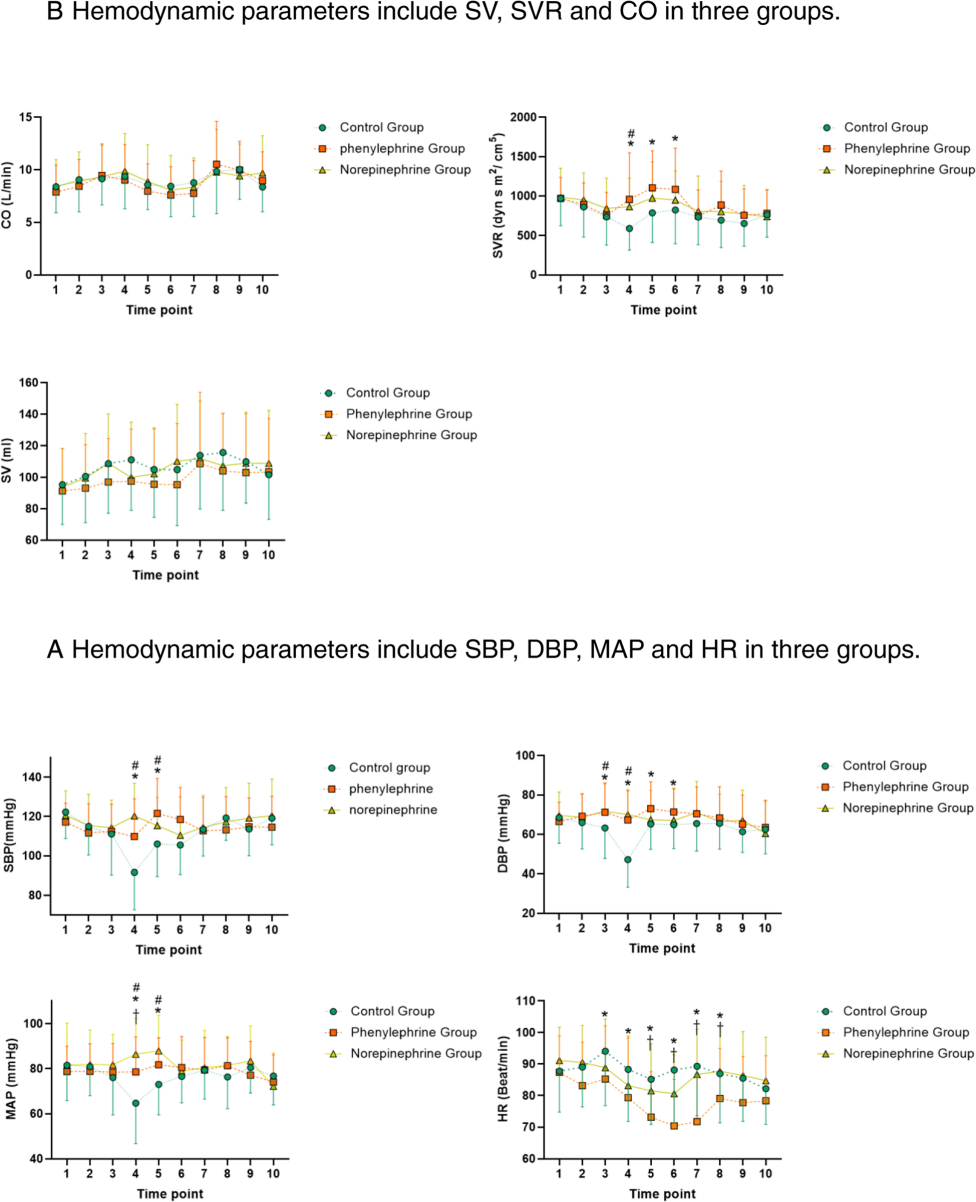
Hemodynamic parameters. **a** Stroke volume (SV), cardiac output (CO), and systemic vascular resistance (SVR) by LiDICOrapid monitoring at the 10 time points. Post hoc Bonferroni correction was performed for within- versus between-subject comparisons. Data are expressed as mean ± standard deviation. *0.25 μg/kg/min phenylephrine vs. control group, *P* < 0.05; ^#^0.05 μg/kg/min norepinephrine vs. control group, *P* < 0.05; †0.25 μg/kg/min phenylephrine vs. 0.05 μg/kg/min norepinephrine group*, P* < 0.05. **b** Fluctuations in heart rate (HR), systolic blood pressure (SBP), diastolic blood pressure (DBP), and mean arterial pressure (MAP) during operation at 10 time points. Markers are means, and error bars are standard deviation. *statistical significance between the 0.25 μg/kg/min phenylephrine and control groups; ^#^statistical significance between 0.05 μg/kg/min norepinephrine group and control groups; ^†^statistical significance between 0.25 μg/kg/min phenylephrine and 0.05 μg/kg/min norepinephrine groups. Post hoc Bonferroni correction was performed for within- versus between-subject comparisons

In Phenylephrine group, the SBP and MAP higher than control group at T4,5 timepoints. DBP In Phenylephrine group were significantly higher than control group at T3,4,5,6 timepoints. HR In Phenylephrine group were significantly lower than control group at T3,4,5,6,7,8 timepoints, and also lower than Norepinephrine group at T5,6,7,8,9 timepoints (Supplement-table-1) (Fig 2a). SVR in Phenylephrine Group significantly were higher than Control group at T4,5,6 timepoints. T4: 957.4 ± 590.3 vs 590.1 ± 273.7 (*P* < 0.000001); T5: 1104 ± 468.0 vs 789.4 ± 376.2 (*P* = 0.000002). T6: 1084 ± 524.8 vs 825.2 ± 428.6 (*P* = 0.000188) (Fig 2b).

In Norepinephrine group, SBP and MAP were higher than control group at T4,5 timepoints. DBP were higher than control group at T3,4 timepoints (Supplement-table-1) (Fig 2a). SVR in Norepinephrine Group was significantly higher than Control group at T4 timepoints. T4: 865.0 ± 360.1 vs 590.1 ± ±273.7 (*P* = 0.000043) (Fig. 2b).

We also proceed inter-group comparison to reflect the variation trend in each group. In control group, compare with baseline, the SBP was significantly decreased at T3, T4, T5, T6, T7 (Supplement-table-1). The DBP was significantly decreased at T4, and the MAP was significantly decreased at T4, T5. In control group, compare with T1(baseline), the CO was significantly increased at T9, the SV was significantly increased at T7, T8 timepoint, and the SVR was significantly decreased at T3, T4,T7, T8,T9,T10 timepoint (Fig. 2b) (Supplement-table-1). In norepinephrine group, compare with T1(baseline), the SBP was slightly decreased at T6, the DBP was significantly decreased at T10 timepoint, the MAP was slightly decreased at T10 and the HR was slightly decreased at T4, T5, T6 timepoint (Supplement-table-1). (Fig. 2a). In phenylephrine group, compare with T1 timepoint (baseline), the HR was slightly decreased at T5, T6, T7, T8, T9, T10 timepoint (Supplement-table-1). The CO was significantly increased at T8, 10.52 ± 4.104 (*P* < 0.0001), T9, 9.965 ± 2.742 (*P* = 0.0003).

### Blood gas indices

**Fig. 3:**
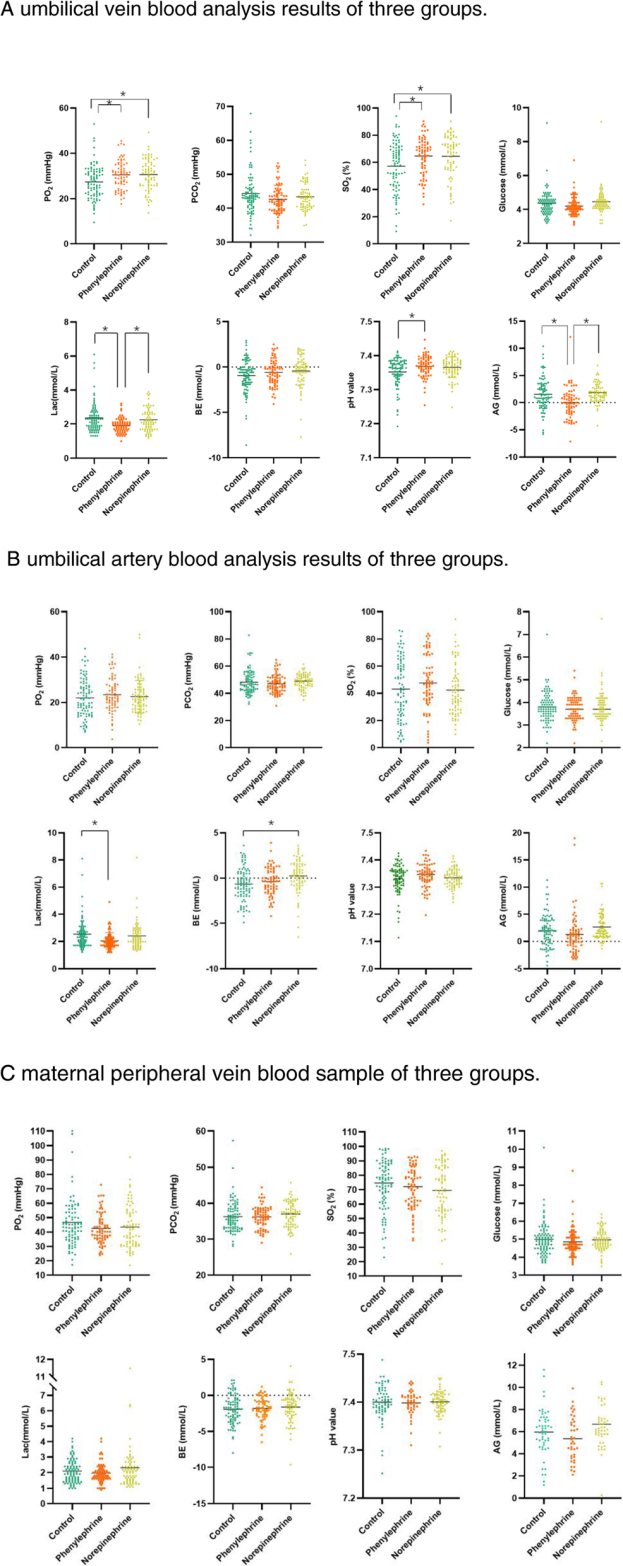
Blood gas analyses. PO_2_, PCO_2_, SO_2_, glucose, lactate (Lac), base excess (BE), pH value, and anion gap (AG) of the (**a**) umbilical vein, **b** umbilical artery, **c** maternal peripheral vein blood sample of the three groups. * *P* < 0.05

The UV PO_2_ in Phenylephrine, 30.50 ± 6.24 (*P* = 0.0143) and norepinephrine, 30.62 ± 6.91 (*P* = 0.0093) significantly higher and SO_2_ values in Phenylephrine, 64.68 ± 13.79 (*P* = 0.0109) and norepinephrine, 64.49 ± 15.76 (*P* = 0.0123) than those in the control group,27.44 ± 7.54; 57.26 ± 17.92. However, the UV lactate level,1.91 ± 0.43 in the phenylephrine group was significantly lower than those in the control, 2.30 ± 0.84 (*P* = 0.0003) and norepinephrine groups 2.25 ± 0.66 (*P* = 0.0106). The UV BE value showed no significant difference among the three groups. The phenylephrine group had a relatively higher UV pH value7.37 ± 0.03 (*P* = 0.0113) than those in the control7.36 ± 0.04, but the mean pH value in all three groups was within the normal clinical range. The UV AG value was significantly lower in the phenylephrine group-0.02 ± 2.73 than those in the control1.49 ± 2.96 (*P* = 0.0005) and norepinephrine groups1.84 ± 1.72 (*P* = 0.0001) (Fig. 3a).

Regarding the UA parameters, there was no significant difference in the PO_2_, SO_2_, PCO_2_, pH, AG, and glucose values among the three groups. The UA lactate level in the phenylephrine group2.05 ± 0.61 (*P* = 0.0038) was significantly lower than that in the control group,2.53 ± 1.01. Only the norepinephrine group showed a positive UA BE value 0.24 ± 1.86when compared with the other two groups-0.53 ± 1.84, − 0.38 ± 1.53 (*P* = 0.0039) (*P* = 0.0056) (Fig. 3b).

Regarding the maternal PV parameters, there were no significant differences in any of the parameters among the three groups (Fig. 3c).

### Comparison of adverse reactions among the three groups

In the phenylephrine group, bradycardia occurred in two cases, but there was no significant difference compared with the other two groups. Administering prophylactic norepinephrine or phenylephrine infusion significantly reduced the incidence of intra-operative hypotension during CD as compared with the control group (phenylephrine vs. control group, χ2 value = 21.04, df = 1, *P* < 0.0001; norepinephrine vs. control group, χ2 value = 24.44, df = 1, P < 0.0001). The phenylephrine group has lower Nausea incidence (phenylephrine vs. control group, χ2 value = 8.088, df = 1, *P* = 0.0045). Control group has relatively higher incidence of intra-operative tachycardia (phenylephrine vs. control group, χ2 value = 7.695, df = 1, *P* = 0.0055; norepinephrine vs. control group, χ2 value = 8.011, df = 1, *P* = 0.0046). When compare each group, after Bonferroni adjustment, the *P* value < 0.0167 indicated the significant different. There was no significant difference in the incidence of vomiting, dizziness, difficult breathing among the three groups (Table 3).

**Table 3:** Maternal outcomes in three groups

Variables	Control group (*n* = 78)	Phenylephrine group (*n* = 71)	Norepinephrine group (*n* = 74)	χ^2^ value	df	*P* value
Bradycardia (n)	0 (0)	0.03 (2)	0.01 (1)	2.22	2	0.329
Tachycardia (n)	0.10 (8)	0 (0) *	0 (0) ^#^	15.43	2	< 0.001
Intraoperative hypotension (n)	0.47 (37)	0.13 (9) *	0.11 (8) ^#^	35.31	2	< 0.001
Nausea (n)	0.14 (11)	0.01 (1) *	0.08 (6)	8.07	2	0.018
Vomiting (n)	0.01 (1)	0 (0)	0.01 (1)	0.9	2	0.624
Difficulty breathing (n)	0.12 (9)	0.03 (2)	0.03 (2)	7.12	2	0.028
Dizziness (n)	0.08 (6)	0 (0)	0.03 (2)	6.61	2	0.037

Data expressed as rate (number);*0.25 μg/kg/min phenylephrine vs. control group, *P* < 0.05; #0.05 μg/kg/min norepinephrine vs. control group, *P* < 0.05; †0.25 μg/kg/min phenylephrine vs. 0.05 μg/kg/min norepinephrine group, *P* < 0.05 based on chi-squared test. (after Bonnfini adjust the *P* value < 0.0167 has the significant different for inter-group comparison)

## Discussion

In this study, we compared the effects of phenylephrine and norepinephrine administered as continuous infusion during elective CD on the maternal hemodynamic parameters and the maternal and fetal outcomes. We determined that phenylephrine and norepinephrine have similar efficacy for the prevention of SA-induced hypotension, with no difference in the incidence of maternal bradycardia. However, phenylephrine better preserved the SVR by maintaining appropriate cardiac afterload and provided better neonatal outcomes base on blood gas parameter.

In phenylephrine group, the SBP remain stable during the whole operation. In T4, T5 timepoint, decreased the SBP, DBP and MAP were observed in control group, but no significant decrease observed in phenylephrine and norepinephrine groups.

In this study, we successfully employed the LiDCOrapid system for noninvasive assessment of the macro-hemodynamic parameters (CO, SV, and SVR) during phenylephrine or norepinephrine infusion in CD. The LiDCOrapid system was previously validated for use in non-pregnant and pregnant populations [15–17]. This system enables continuous assessment of the SV based on noninvasive pulse contour analysis under spontaneous breathing, which provides a reliable hemodynamic trend [18, 19]. The typical hemodynamic response to SA in parturients adversely affects the SVR, a precise dynamic marker of preload responsiveness, and requires a compensatory antagonist [20]. Phenylephrine increases the SVR to counteract the SA-induced vasodilatation. In our study, the SVR in the phenylephrine group was significantly higher than that in the other two groups at T4, T5, and T6 time points.

Based on our results, both 0.25 μg/kg/min phenylephrine and 0.05 μg/kg/min norepinephrine infusions maintain sufficient CO. Unlike norepinephrine, which has a weak β-agonistic action, phenylephrine has no β-agonistic action and is expected to cause a greater decrease in HR. The decrease in HR caused by phenylephrine may affect the maternal CO. [21] In our result, the HR of phenylephrine group decreased compare with the baseline, and slightly lower than control group, but also in the clinical normal range. Even though, the CO maintain stable during the whole operation. The physiologic principal due to the SVR increase in phenylephrine relatively compensate the HR decrease, then maintain the CO level. In Nagankee’s study, the higher dose of phenylephrine (0.5 μg/kg/min) caused lower CO. [22] Phenylephrine negatively affects the CO in a dose-independent manner [23]. However, in this study, there was no significant decrease in the CO in the phenylephrine group, likely due to the appropriate dosage (0.25 μg/kg/min) in our research chosen. The same as CO, the maternal SV also remained constant during phenylephrine infusion throughout the study period.

In the present study, phenylephrine and norepinephrine significantly increased the PO_2_ and SO_2_ values in the UV. These parameters are known to correlate with fetal oxygenation. Stewart et al. emphasized that even with fetal compromise, there is a need to maintain fetal oxygen delivery [8]. The increase in the UV PO_2_ and SO_2_ values indicates that phenylephrine and norepinephrine enable greater oxygen delivery to the fetus. The changes in the UV glucose levels noted in this study during the vasopressor infusions reflected the changes in the maternal blood glucose levels due to stress reaction. However, the norepinephrine infusion could also have exhibited the stress hormone effect, increasing the UV glucose levels [24]. In the present study, neither the UV nor the UA glucose levels varied among the three groups, which indicates that, at the appropriate dosage, both phenylephrine and norepinephrine can maintain the parturient and the fetus in a low-stress condition. Serum lactate level is the best surrogate indicator of metabolic changes in the fetus. The main finding of our study is that both phenylephrine and norepinephrine tended to decrease the UV lactate levels. The UV lactate level was the lowest in the phenylephrine group, suggesting that phenylephrine could improve the umbilical blood flow and thereby decrease the metabolic products level, further improving the fetal circulation and oxygen supply. Phenylephrine has the propensity to increase the afterload owing to its α-antagonist action. Catecholamines do not readily cross the placental barrier [25]; hence, the UA blood gases cannot be affected by phenylephrine or norepinephrine. Changes in the UA blood gases are more likely the result of the fetal stress and fetal catecholamine level per se. Such changes affect the UA pH value. In this study, the UA pH value was better in the phenylephrine group than in the norepinephrine. Thus, infusion of low-dose phenylephrine allows for better UA pH. In contrast, norepinephrine induces β-agonist-mediated stimulation of the fetal metabolism, leading to slight reduction in the UA pH value. Ngan Kee et al. reported no difference in the UA pH value when comparing phenylephrine with norepinephrine, which is consistent with our results. BE is a widely used indicator of fetal distress because higher BE values indicate better fetal acid-base status with reduced incidence of fetus acidosis. We observed higher BE values with the use of phenylephrine than those in the control or norepinephrine groups. The lower number of episodes of maternal/fetal acidosis in the phenylephrine group may reflect the positive effects of phenylephrine on the fetus.

In our study, Prophylactic norepinephrine or phenylephrine infusion effectively reduce incidence of tachycardia and intraoperation hypotension during CD. Allen et al. reported a rate of incidence of hypotension of 15% with 50 μg/min of phenylephrine, which is similar to our results. Nausea, occur secondary to cerebral hypoperfusion due to hypotension [10, 26]. Numerous studies have reported reduced incidence of hypotension, intraoperative nausea, vomiting, and dizziness with prophylactic bolus of phenylephrine or norepinephrine at various doses [8, 27, 28]. In present study, nausea was lower in phenylephrine group.

A few study limitations need to be considered. We did not analyze the metabolic effect of parturients and the neonatus after delivery 24 h in the different vasopressor groups. Thus, although the administration of phenylephrine for prophylaxis of post-SA hypotension has shown promising results, further research is required to explore the further metabolic effect.

## Conclusion

In summary, Phenylephrine, in particular, better preserves the SVR and remain appropriate blood pressure. However, the fetus outcomes are better with phenylephrine than with norepinephrine infusion, as evaluated by the decrease in the UV lactate and the increase in the UV pH value. Therefore, continuous infusion of phenylephrine at 0.25 μg/kg/min may improve the outcomes of parturients. These results suggested that the use of low-dose phenylephrine (0.25 μg/kg/min) does not decrease the CO, thereby providing a better SVR and better perfusion condition of the fetus.

## Supplementary information

**Additional file 1 **: **Supplement Table 1**. SBP. **Supplement Table 2**. DBP. **Supplement Table 3**. MAP. **Supplement Table 4**. HR. **Supplement Table 5**. SVR. **Supplement Table 6**. CO. **Supplement Table 7**. SV

## Data Availability

The raw data of this study are available from the corresponding author on reasonable request.

## Abbreviations

SA: Spinal anesthesia
CD: Cesarean delivery
UV: Umbilical vein
UA: Umbilical artery
PV: Peripheral vein
SBP: Systolic blood pressure
SVR: Systemic vascular resistance
CSEA: Combined spinal-epidural anesthesia
BP: Blood pressure
HR: Heart rate
IV: Intravenous
ASA: American Society of Anesthesiologists
LR: Ringer’s solution
SV: Stroke volume
CO: Cardiac output
DBP: Diastolic blood pressure
MAP: Mean arterial pressure
PO2: Oxygen partial pressure
SO2: Oxygen saturation
PCO2: Carbon dioxide partial pressure
BE: Base excess
AG: Anion gap
