# Supplementary material for: Effects of continuous infusion of phenylephrine vs. norepinephrine on parturients and fetuses under LiDCOrapid monitoring: a randomized, double-blind, placebo-controlled study
Source: BMC Anesthesiol. 2020 Sep 7;20:229. doi: 10.1186/s12871-020-01145-0 (PMC7487484; doi:10.1186/s12871-020-01145-0)
Supplement: Supplementary file 1 — Additional file 1 : Supplement Table 1. SBP. Supplement Table 2. DBP. Supplement Table 3. MAP. Supplement Table 4. HR. Supplement Table 5. SVR. Supplement Table 6. CO. Supplement Table 7. SV [file 12871_2020_1145_MOESM1_ESM.docx]

1. Supplement table -SBP

| SBP | Control Group | Phenylephrine Group | Phenylephrine vs Control, P value | Norepinephrine Group | Norepinephrine vs Control, P value |
| --- | --- | --- | --- | --- | --- |
| T1 | 122.5±13.63 | — | — | — | — |
| T3 | 110.4±20.67* | — | — | — | — |
| T4 | 92.33±19.30^#^ | 109.8±19.09 | P=0.002697 | 120.3±16.60 | P <0.000001 |
| T5 | 106.0±15.66^☆^ | 121.6±17.88 | P=0.016501 | 115.4±14.33 | P=0.002804 |
| T6 | 105.9±15.30^※^ | — | — | — | — |
| T7 | 113.7±13.33^†^ | — | — | — | — |

^*^In control group, T3 vs T1, P <0.0001. ^#^ In control group,T4 vs T1, P <0.0001.^☆^In control group,T5 vs T1, P <0.0001.^※^In control group,T6 vs T1, P <0.0001.^†^In control group,T7 vs T1, P =0.0050.

2. Supplement table -DBP

| DBP | Control Group | Phenylephrine Group | Phenylephrine vs Control, P value | Norepinephrine Group | Norepinephrine vs Control, P value |
| --- | --- | --- | --- | --- | --- |
| T1 | 68.49±13.05 | — | — | 69.54±11.98 | — |
| T3 | 63.21±15.44 | 71.2314.86 | P=0.002474 | 71.80±14.86 | P=0.001132 |
| T4 | 47.18±13.98^*^ | 67.30±14.72 | P<0.000001 | 70.00±14.72 | P <0.000001 |
| T5 | 65.26±12.91 | 73.01±13.69 | P=0.003311 | — | — |
| T6 | 64.87±12.19 | 71.39±11.99 | P=0.020635 | — | — |
| T10 | — | — | — | 60.42 ±16.50 ^#^ | — |

^#^ In control group, T4 vs T1, P <0.0001; ^&^In norepinephrine group, T10 vs T1, P =0.0011

1. Supplement table -MAP

| MAP | Control Group | Phenylephrine Group | Phenylephrine vs Control, P value | Norepinephrine Group | Norepinephrine vs Control, P value | Norepinephrine vs Phenylephrine, P value |
| --- | --- | --- | --- | --- | --- | --- |
| T1 | 81.34±16.03 | — | — | 81.59±18.59 | — | — |
| T4 | 64.96±18.44 | 78.52±15.61 | P<0.000001 | 86.39±15.97 | P<0.000001 | P=0.009866 |
| T5 | 73.01±14.02 | 81.75±11.94 | P=0.001162 | 87.95±15.78 | P<0.000001 | — |
| T10 | — | — | — | 72.15±14.79 | — | — |

^#^ In control group, T4 vs T1, P <0.0001. ^☆^ In control group, T5 vs T1, P =0.0029, ^&^In norepinephrine group, T10 vs T1, P=0.0016

1. Supplement table -HR

| HR | Control Group | Phenylephrine Group | Phenylephrine vs Control, P value | Norepinephrine Group | Norepinephrine vs Phenylephrine, P value |
| --- | --- | --- | --- | --- | --- |
| T1 | — | 87.41±11.57 | — | 91.16±10.54 | — |
| T3 | 94.13± 17.43 | 85.27±16.87 | P=0.001804 | — | — |
| T4 | 88.59±15.98 | 79.31±19.55 | P=0.001077 | 83.19±15.07^&^ | — |
| T5 | 85.12±14.46 | 73.14±14.47* | P=0.000009 | 81.54±14.91^$^ | P=0.002737 |
| T6 | 87.37±16.63 | 70.41±14.74 ^#^ | P<0.000001 | 80.65±12.33^△^ | P=0.000177 |
| T7 | 88.67±15.56 | 71.72±13.93^☆^ | P<0.000001 | 86.73±14.32 | P<0.000001 |
| T8 | 86.72±15.60 | 79.08±15.88^※^ | P=0.007320 | 87.72±13.84 | P=0.002530 |
| T9 | 86.34±13.74 | 77.79 ±14.61† | P=0.002531 | 86.41±13.94 | P=0.002530 |
| T10 | — | 78.34±14.35‡ | — | — | — |

*In Phenylephrine group, T5 vs T1, P <0.0001; ^#^ In Phenylephrine group, T6 vs T1, P <0.0001;^☆^In Phenylephrine group, T7 vs T1, P <0.0001;^※^In Phenylephrine group, T8 vs T1, P =0.0084; †In Phenylephrine group, T9 vs T1, P=0.0013; ‡In Phenylephrine group, T10 vs T1, P=0.0030

^&^In norepinephrine group, T4 vs T1, P=0.0034; ^$^ In norepinephrine group, T5 vs T1, P=0.0002; ^△^In norepinephrine group, T6 vs T1, P<0.0001

1. Supplement table -SVR

| SVR | Control Group | Phenylephrine Group | Phenylephrine vs Control, P value | Norepinephrine Group | Norepinephrine vs Phenylephrine, P value |
| --- | --- | --- | --- | --- | --- |
| T1 | 970.8±344.9 | — | — | — | — |
| T3 | 738.2±358.3* | — | — | — | — |
| T4 | 590.1±273.7^#^ | 957.4±590.3 | P<0.000001 | 865.0±360.1 | P=0.000043 |
| T5 | 789.4±376.2 | 1104±468.0 | P=0.000002 | — | — |
| T6 | 825.2± 428.6 | 1084±524.8 | P=0.000188 | — | — |
| T7 | 735.3±350.0^☆^ | — | — | — | — |
| T8 | 696.5±348.3^※^ | — | — | — | — |
| T9 | 654.9±289.7† | — | — | — | — |
| T10 | 766.5±286.6‡ | — | — | — | — |

*In control group, T3 vs T1, P =0.0003;^#^In control group, T4 vs T1, P <0.0001;^☆^In control group, T7 vs T1, P =0.0002;^※^In control group, T8 vs T1, P <0.0001;†In control group, T9 vs T1, P <0.0001; ‡In control group, T10 vs T1, P=0.0022

1. Supplement table -CO

| CO | Control Group | Phenylephrine Group |
| --- | --- | --- |
| T1 | 8.381±2.451 | 7.868±2.578 |
| T8 | — | 10.52±4.104^&^ |
| T9 | 10.03±2.849 * | 9.965±2.742^$^ |

*In control group, T9 vs T1, P =0.0033; ^&^ In Phenylephrine group, T8 vs T1, P<0.0001; ^$^In Phenylephrine group,T9 vs T1,P =0.0003

1. Supplement table -SV

| SV | Control Group |
| --- | --- |
| T1 | 95.41±25.33 |
| T7 | 110.3±31.56* |
| T8 | 104.9±30.35^#^ |

*In control group, T7 vs T1, P =0.0026; ^#^In control group, T8 vs T1, P =0.0010
